# Study of the Catalytic Activity of the Compounds Hydrotalcite Type Treated by Microwave in the Self-Condensation of Acetone

**DOI:** 10.1155/2021/1551586

**Published:** 2021-12-23

**Authors:** Jamal Houssaini, Mohammed Naciri Bennani, Hamid Ziyat, Said Arhzaf, O. Qabaqous, Abdellatid Amhoud

**Affiliations:** LCBAE, Laboratory of Chemistry and Biology Applied to the Environment, MAC, Research Team “Materials and Applied Catalysis”, Chemistry Department, Moulay Ismail University, B.P. 11201 Zitoune, Meknes 50000, Morocco

## Abstract

The self-condensation reaction of acetone, producing diacetone alcohol (DAA), is of great industrial importance. It was used to study the catalytic activity of Mg-Al catalysts synthesized by the coprecipitation method. For this purpose, we synthesized Mg-Al based hydrotalcite with a molar ratio of 3, obtained either after conventional heating or after microwave irradiation with of 100 W for three minutes. Structural and chemical properties of the obtained catalysts were characterized, using different techniques: X-ray diffraction (XRD), Fourier transform infrared spectra (FTIR), scanning electron microscope (SEM), equipped with energy dispersive X-ray (EDX), and specific surface area of the catalysts were determined by the methylene blue (MB) adsorption method. Also, these catalysts were tested in the self-condensation reaction of acetone at 273 K in the liquid phase without solvent, a reaction which requires very high catalytic activity. The microwave treatment improves the catalyst activity, and the conversion of acetone to diacetone alcohol increases from 13.2 to 18.3% after 8 h of reaction. Moreover, the microwave-treated hydrotalcite catalyst, calcined at 723 K and rehydrated under a flow of N_2_, is the most active and gives conversion of acetone of 52% under the same reaction conditions.

## 1. Introduction

Several important industrial reactions such as condensation, hydrogenation, dehydrogenating reactions, oxidation, alkylation, and addition are carried out in the homogeneous phase by the liquid bases, for instance, KOH, NaOH, and Na_2_CO_3_. Heterogeneous catalysis, unlike to this point mostly used homogeneous catalysis, brings advantages, like higher selectivity, easier catalyst recovery, elimination of wastewater streams, and by-products that might contribute to pollution of the environment [1]. Hydrotalcites (HT), which are layered double hydroxides (LDHs) family, are well-known as heterogeneous base catalysts for various reactions. They need layered structures containing anions and H_2_O molecules between charged metal hydroxides with the chemical formula:

(1) $$\begin{array}{c} {\left[{\text{M}}_{1-\text{x}}^{2+}M_{\text{x}}^{3+}{\left(\text{OH}\right)}_{2}\right]}^{\text{X}+}{\left[{\text{A}}_{\text{x}/\text{n}}^{\text{n}-}\cdot {\text{mH}}_{2}\text{O}\right]}^{\text{x}-}, \end{array}$$

where M^2+^ (divalent cations = Mg, Zn, Ni, Cu, and Mn) and M^3+^ (trivalent cations = Al, Cr, Co, Mn, and Fe); A is the exchangeable anions like OH^−^, Cl^−^, NO_3_^−^, CO_3_^2−^, and SO_4_^2−^. Particularly, Mg–Al hydrotalcites, where M^2+^ and M^3+^ sites comprise Mg and Al, are representative hydrotalcites [2, 3]. The structure of hydrotalcite Mg_6_Al_2_(OH)_16_CO_3_.4H_2_O resembles that of brucite Mg(OH)_2_. Within the latter structure, the magnesium cations are octahedrally coordinated by hydroxyl ions, leading to the stacks of edge-shared layers of the octahedral. For the HT structure, a part of the Mg^2+^ ions is replaced by Al^3+^ ions leading to positive charge layers, whose charge is compensated by interlayer anions, essentially the carbonate ions. Furthermore, water molecules are present within the interlayer [4]. To create catalytic activity in aldol condensation reactions, HT should undergo a selected treatment. For gas-phase condensation reactions at a hot temperature, calcination at 723 K is sufficient [5]. The resulting Mg-Al-(O)_x_ mixed oxide is capable of catalyzing various reactions just like the self-condensation of acetone exhibiting strong Lewis basicity [5] and therefore the condensation of formaldehyde and acetone [6].

**Scheme 1:**
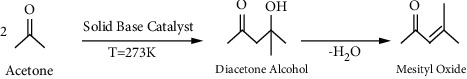
Self-condensation of acetone.

To boost the catalytic activity at lower temperatures, the calcined samples must be rehydrated under inert atmosphere without carbonate of air. This treatment provides the restoration of the layered structure and formation of the meixnerite with OH^−^ ions within the interlayer domain. A useful and comparatively well-described condensation reaction within the liquid-phase using rehydrated calcined HT is the self-condensation of acetone yielding diacetone alcohol (Scheme 1) [7]. It may be concluded that a reaction temperature of 273 K is advantageous to prevent the dehydration of DAA to mesityl oxide and thus to avoid the liberation of water which could influence the accessibility and the reactivity of the catalyst.

## 2. Materials and Methods

### 2.1. Materials

Acetone (C_3_H_6_O; purity = 99%) was purchased from Prolabo Chemicals. The magnesium chloride (Mg(Cl)_2_·6H_2_O; purity = 98.9%), aluminum chloride (Al(Cl)_3_·6H_2_O; purity = 99.1%), sodium carbonate (Na_2_CO_3_; purity = 99%), and sodium hydroxide (NaOH; purity = 99%) were purchased from LOBA Chemie for the synthesis of hydrotalcite samples. The distilled water was used in all experiments.

### 2.2. Synthesis

The coprecipitation method is that the foremost typically will not synthesis LDHs. It consists of the simultaneous precipitation of aqueous solutions of soluble metal salts containing the anion which will be incorporated into the structure [8]. The anion can proceed from the metallic salt or from the essential solution accustomed to produce the precipitation of the metals. This method allows the direct preparation of LDHs with an oversized type of interlamellar anions. The hydrotalcite samples with Mg-Al molar ratio of three were synthesized by the coprecipitation method, pH was maintained between 9 and 10, and prepared from gels produced by mixing two solutions: solution A containing 0.15 moles of Mg(Cl)_2_·6H_2_O and 0.05 moles of Al(Cl)_3_·6H_2_O dissolved in 150 mL of distilled water with Mg/Al = 3 and solution B containing 1.8 moles of NaOH and 0.2 moles of Na_2_CO_3_ dissolved within the identical volume of the answer A. Both solutions are added at a rate of 1 mL/min under a full of mechanical stirring at ambient temperature. After adding the two solutions, the precipitate was obtained and divided into two fractions:

The first one was treated by microwaves with a power of 100 W during 3 minutes, and the second was refluxed at 338 K for 18 h to allow crystal growth. The precipitate formed is filtered and washed thoroughly with quandary until the pH of the filtrate was 7. Washed precipitate was dried in an oven at 353 K for 24 h and ground to convert it into powder form; the solids obtained were named HT3-MW and HT3, respectively.

### 2.3. Exchange of Chloride Anions

2 g of solid was suspended in a 0.5 M solution of sodium carbonate (100 mL) and stirred at 343 K for two hours. After filtration, the solid was washed several times until the solution was free of chloride ions (AgNO_3_-test) and dried at 353 K. The solids obtained were named HT3-MW-E and HT3-E, respectively.

The solids syntheses HT3-MW-E and HT3-E were first activated by calcining to 723 K in a flow of air. The temperature was raised at 723 K and maintained for 12 h. After returning to room temperature, the solid is immediately used in the reaction. Rehydration of calcined hydrotalcite was carried at room temperature under a flow of N_2_ gas (50 mL/min) saturated with water vapor for 12 h.

### 2.4. Characterization of the Catalysts

X-ray diffraction measurements were made on a Philips (PW-1800) diffractometer (Cu-K*α* = 1.5418 Å), radiation provided by a graphite monochromator. The X-ray source is powered by a current of 40 kV for a current of 20 mA. The spectra of the various samples were recorded during a 2*θ* range between 5° and 70° with an angular increment of 0.04°, and therefore, the sweep speed is 0.08°/min. The catalysts were as pellets consisting of 4 mg of sample dispersed in 96 mg of KBr. The FT-IR spectra were recorded from 4000 to 400 cm^−1^, with a resolution of 4 cm^−1^ by a Shimadzu type instrument (IRAffinity-1S), equipped with a triglycine sulfate (TGS) detector.

The surface morphology of the catalysts was conducted by scanning electron micrographs (SEM), equipped with energy dispersive X-ray (EDX) analysis. The images of the catalysts were taken with a scanning electron microscope (model: JSM-IT500 HR), at a magnification of about 3000 times. The accelerating voltage of about 10 kV was applied to avoid the surface charging. There are different techniques to measure the specific surface of solids. The adsorption of gases (i.e., the condensation of molecules on the surface of solids) allows determining the specific surface from the relation between the applied pressure and the volume of adsorbed gas. Another technique used in this work is the adsorption of molecules from a solution on a solid surface, in particular dyes such as methylene blue which adsorbs on negatively charged clay surfaces. Therefore, the specific surface area of the particles can be determined by the amount of methylene blue adsorbed.

### 2.5. Reaction Procedure

15 mL of acetone was taken into a two-necked 50 ml flask and cooled to 273 K with ice. After 10 minutes of cooling, the freshly activated catalyst 0.5 g was introduced. Progress of the reaction was monitored in terms of the consumption of acetone. Analysis of product mixture was dispensed by gas chromatography (Shimadzu-2010). The GC incorporates a 5% diphenyl and 95% dimethylsiloxane universal capillary column (60 m length and 0.25 mm diameter) and a flame ionization detector (FID).

The experiments were applied under identical reaction conditions to make sure the reproducibility of the reaction. For kinetic measurements, at this effect, a fraction of 30 *μ*l was taken from the reaction mixture at different times using one syringe and diluted with 30 *μ*l cyclohexane, to which 60 *μ*l of an answer has been added decane 0.1 M used as a typical. A series of kinetic experiments were carried by injecting 0.1 *μ*l of every fraction of the compounds into the GC column, out for the determination of the rates. The conversion was calculated by the subsequent formula:

(2) $$\begin{array}{c} \text{Conversion}\left(\%\right)=\frac{{\left(\text{Moles of acetone}\right)}_{\text{reacted}}}{{\left(\text{Moles of acetone}\right)}_{\text{fed}}}\times 100. \end{array}$$

## 3. Results and Discussion

### 3.1. Catalysts Characterization

**Figure 1:**
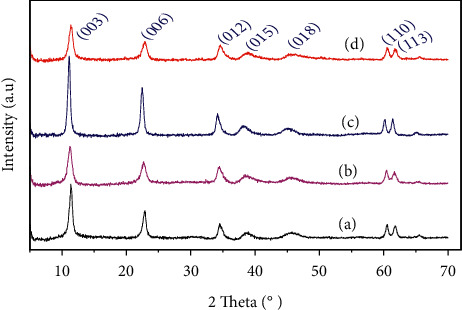
X-ray diffraction patterns of (a) HT3, (b) HT3-MW, (c) HT3-E, and (d) HT3-MW-E.

The X-ray diffraction patterns of hydrotalcite samples are shown in Figure 1.

The catalysts show crystalline hydrotalcite patterns. All the diffraction peaks are in good agreement with the characteristics of the hexagonal phase as the (003), (006), and (012) peaks and indicate the formation of the compounds with a good crystallinity and no impurities (Figure 1) [9]. The building block parameters were calculated assuming a 3R stacking sequence, that is, *a* = 2*d*_110_ and *c* = 3*d*_003_. Accordingly, the (c) parameter is the interlayer thickness and is regulated by water content together with the amount, size, orientation, and charge of the anions located between the brucite-like layers. The cell parameter (a) corresponds to the everyday cation–cation distance inside the brucite-like layers [10].

These parameters of the synthesized hydrotalcite are calculated by knowing the reticular planes (*hkl*) diffraction peaks from the Bragg reflection, and thus, the interreticular distance *d*(*hkl*) is given by the following relation [11], and these parameters are given in Table 1.

(3) $$\begin{array}{c} \frac{1}{d_{hkl}^{2}}=\frac{4\left(h^{2}+hk+k^{2}\right)}{3a^{2}}+\frac{l^{2}}{c^{2}}. \end{array}$$

The values of parameters (a) and (c) obtained (Table 1) are closer to those reported in the literature [12]. For all solids, parameter (a) remains virtually unchanged. However, (c) parameter for HT3-E and HT3-MW-E is, respectively, smaller than that of HT3 and HT3-MW. This can be explained by the load effect after anion exchange Cl^−^ by CO_3_^2−^, which generated an excess of negative load in the interfoliar space favoring an electrostatic attraction between carbonate ions and loaded sheets positively, and as a result, there is a decrease in interleaf distance after exchange [12]. The Debye–Scherrer method is a crystallographic analysis technique based on X-ray diffraction and used to determine the average size of crystalline materials, according to the following formula [13]:

(4) $$\begin{array}{c} L=\frac{K\cdot \lambda }{\beta }\times \frac{1}{\cos\left(\theta \right)}, \end{array}$$

where *K* is a constant related to crystallite shape, normally taken as 0.89, *λ* is the X-ray wavelength in (Å), and *β* is the width of the diffraction peak at half maximum height in radians. The theta can be in degrees or radians. Mean crystallite size estimation obtained for the different hydrotalcite synthesis is given in Table 2.

**Figure 2:**
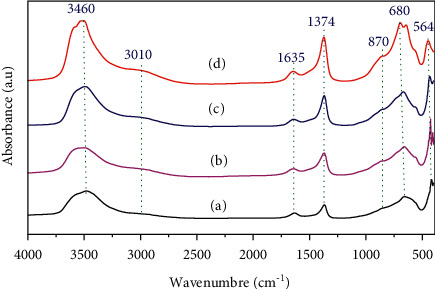
FT-IR spectra of (a) HT3, (b) HT3-MW, (c) HT3-E, and (d) HT3-MW-E.

According to the values given in Table 2, it can be seen that for all the samples synthesized, the average particle size is in the nonmetric range. This size increases after the exchange of chlorides Cl^−^ by carbonates CO_3_^2−^ for catalysts treated or not treated by microwave. However, samples treated by microwaves have a lower average particle size than those of solids that have undergone conventional heating at 338 K. This suggests an increase in the specific surface area and may be an increase in catalytic activity, which will be checked in the study part of the condensation reaction. The synthesized samples were also analyzed by infrared spectroscopy to verify the main existing molecular groupings and vibration types between the atoms. FT-IR spectra of the as-prepared samples are shown in Figure 2. The FT-IR spectrum of the various hydrotalcite is similar to those published elsewhere [14].

Peak in the range of 3450–3550 cm^−1^ was attributed to the stretching vibrations of physisorbed water, structural hydroxyl groups in the brucite-like layer [14], and/or the valence vibration mode of the OH-(M-OH) of divalent and trivalent cations (Mg^2+^ and Al^3+^) [15]. A shoulder at 3010 cm^−1^ was assigned to hydrogen bonding between water molecules and the interlayer carbonate anions. The bands at 1635 and 1374 cm^−1^ showed the presence of interlayer water molecules and carbonate anions, respectively [16]. The low-frequency region showed a band at about 870 cm^−1^ which is characteristic for the out-of-plane deformation of carbonate, whereas the in-plane bending is located at 680 cm^−1^ [16]. The band at 564 cm^−1^ corresponding to the translation modes of hydroxyl groups is influenced by Al^3+^ cations [17].

**Figure 3:**
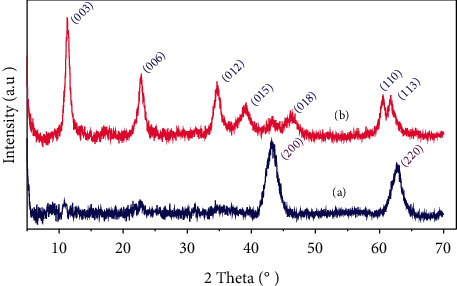
X-ray diffraction powder patterns of (a) HT3-MW-Ec and (b) HT3-W-EcR.

From the spectra obtained (Figure 2), it is found that the characteristic bands of the carbonate species located at 1374 cm^−1^ are more intense for the samples exchanged (HT3-E and HT3-MW-E) compared to those observed for the samples not exchanged (HT3 and HT3-MW), indicating the insertion of CO_3_^2−^ ions into the interfoliar space, and the replacement of chloride ions from the starting salts and not removed after washing. The hydroxylation treatment was carried out on the calcined sample at 723 K. The X-ray diffraction diffractograms of the two catalysts HT3-MW-Ec and HT3-MW-EcR are shown in Figure 3.

The diffractogram of the calcined sample (HT3-MW-Ec) at 723 K (Figure 3) shows the disappearance of peaks (003), (006), and (012) of the lamellar structure, and therefore, the appearance of recent peaks at 2*θ* = 44° and 2*θ* = 63° characteristics the formation of the mixed oxide Mg-Al-(O)_x_. However, the X-ray diffraction spectrum of the rehydroxylated sample (HT3-MW-EcR) shows the reappearance of all peaks (003), (006), and (012) characteristics of the lamellar structure (Figure 3). Therefore, the rehydroxylation treatment transforms the mixed oxide into a lamellar compound having a well-organized structure. This treatment reveals the reconstitution of the lamellar structure of hydrotalcite compounds type and also the formation of the meixnerite phase Mg_6_Al_2_(OH)_18_·4H_2_O [18].

**Figure 4:**
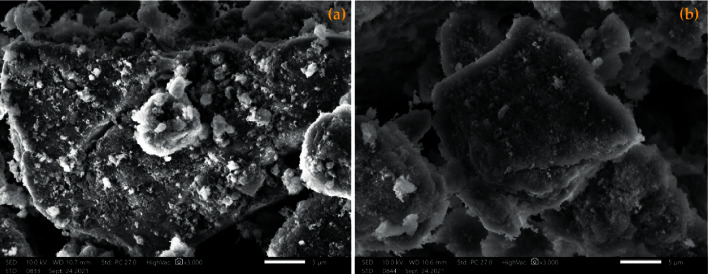
SEM images of (a) HT3-Ec and (b) HT3-MW-Ec.

SEM images were recorded to observe the effect of the synthesis method on the morphology of the catalysts. For this purpose, we compared the morphology of the two catalysts prepared by two methods, either by the conventional heating method or by the microwave irradiation method. SEM images of the two catalysts calcined at 723 K (HT3-Ec and HT3-MW-Ec) are shown in Figure 4.

**Figure 5:**
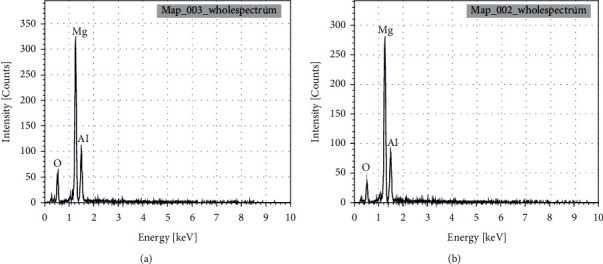
EDX analysis of (a) HT3-Ec and (b) HT3-MW-Ec.

The micrographs of the two solids show well-developed layers with a platelet structure (Figure 4). The morphology of the two mixed oxides obtained by calcination at 723 K is similar to that of the calcined double lamellar hydroxides [19, 20]. However, the microwave-treated catalyst indicates a homogeneous structure with much smaller platelet sizes than those obtained by the conventional heating method, with a larger pore surface. These results are in good agreement with crystallite size calculated by the Debye–Scherrer method (Table 2). The results obtained by energy dispersive X-ray for the two calcined catalysts are shown in Figure 5.

The EDX results of the two analyzed catalysts show that the elements (oxygen, magnesium, and aluminum) characteristics of the mixed oxides are almost identical (Figure 5), with a small difference in the percentage of these elements of about 1–5%. The results of the elemental analysis are given in Table 3.

The specific surface of the solids was calculated by the methylene blue (MB) adsorption method [21]. This technique gives good results compared to the BET method [22]. For this purpose, we use this method to measure the specific surface of the catalysts.

The experimental protocol used is the following: a methylene blue solution was prepared by mixing 1 g of dry MB powder with 200 mL of deionized water. Then, 5 g of calcined catalyst was mixed with 15 mL of deionized water. Then, the MB solution was added to this solution by addition of 1 mL. After each addition of 1 mL of MB, the suspension was mixed by a magnetic stirrer for two minutes. A drop was taken from the solution and placed on Fisher brand filter paper. If the unadsorbed MB formed a permanent blue halo around the aggregate spot of the solid, the end point was reached. Therefore, the specific surface area of the particles can be determined from the amount of MB adsorbed, when all sites of the solid are covered by MB molecules. At this optimal point, the SSA is computed by the following equation [23]:

(5) $$\begin{array}{c} \text{SSA}=\frac{m_{\text{MB}}}{319.86}\times \frac{1\cdot N}{200}\times \frac{A_{N}A_{\text{MB}}}{m_{s}}. \end{array}$$

In equation (5), *m*_MB_ is the mass of the MB, *N* is the number of MB increments added to the suspension solution, *m*_s_ is the mass of the solid, *A*_N_ is Avogadro's number (6.02 × 10^23^ mol^−1^), and *A*_MB_ is the surface area covered by one molecule of methylene blue which is generally considered to be 130 Å^2^. The chemical formula of MB is C_16_H_18_ClN_3_S, with a corresponding molecular weight of 319.86 g·mol^−1^. The obtained SSA results for the two solids are given in Table 4.

These results show that the specific surface area value of the catalyst obtained by the microwave irradiation method (HT3-MW-Ec) is higher than that prepared by the conventional heating method (HT3-Ec). Therefore, the HT3-MW-Ec catalyst has a more ordered structure than HT3-Ec. This is confirmed by the analytical results obtained by scanning electron microscopy (Figure 4).

### 3.2. Catalytic Activity

The catalytic activity of the catalysts was investigated for the formation of the diacetone alcohol by the aldol condensation of acetone. Initially, the reaction was disbursed with the unmodified (HT3 and HT3-MW) catalysts, and these solids showed no activity. It is generally known that chlorides poison the reaction and reduce catalytic activity [18], which is why, within the course of the work; the reactions are conducted with the catalysts that have undergone the exchange by the carbonates. After 8 h of reaction time, a small quantity of DAA is obtained giving a conversion rate of about 0.2%, indicating that these modified catalysts had low catalytic activity. This will be explained by their low basicity, which led us to check the calcined catalysts, and the results obtained with calcined catalysts are given in Table 5.

It was found that calcination improves the catalytic activity of the catalysts, with an increase in the conversion for the microwave-treated calcined catalyst (HT3-MW-Ec), which shows a high conversion of 18.3% after 8 h of reaction, compared to calcined and not irradiated (HT3-Ec), which gives a conversion of 13.2% (Table 5). The best conversion was obtained using the microwave-treated calcined catalyst at 723 K for the self-condensation of acetone.

### 3.3. Influence of Rehydration

**Figure 6:**
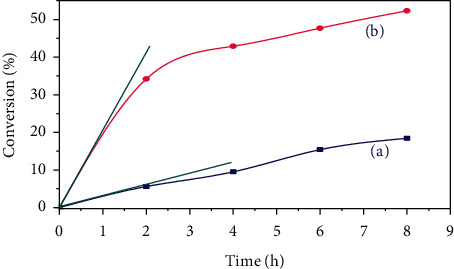
Percentage conversions of acetone with the catalysts: (a) HT-MW-Ec and (b) HT3-MW-EcR, as a function of time.

To examine the effect of the rehydroxylation treatment on the conversion of acetone at 273 K, we tested the catalyst HT3-MW-Ec, which produced the highest conversion (18.3%). The conversion rates deducted after each 8 h of reaction are shown in Figure 6.

There is a significant increase in conversion rates with the catalyst treated by microwaves calcined and rehydroxylated, with a maximum conversion of about 52% after 8 h of reaction (Figure 6). This can be explained by the formation of the news sites as Bronsted type following the reconstruction of the sheet structure of the mixed oxide and the formation of the meixnerite phase. Therefore, the presence of new basic Bronsted sites of OH^−^ type is favorable to the self-condensation of acetone under the reaction conditions used [18].

### 3.4. Initial Rate of Formation of DAA

The evolution of the catalytic activity of the catalysts treated by the microwave (HT3-MW-Ec and HT3-MW-EcR) was also followed by the determination of the initial speeds of the reaction. The latter was determined from the slope of the tangent at the origin of the curves representing the evolution of the conversion rate (%) as a function of time (Figure 5) by the following formula:

(6) $$\begin{array}{c} V_{0\;}=C_{0}\times \;\frac{\text{d}\tau \;}{\text{d}t}. \end{array}$$

The values of the initial rate for the two catalysts are given in Table 6.

All the activated hydrotalcites (thermally treated and rehydrated) were active, and they present much higher catalytic activity for the conversion of acetone to diacetone alcohol. The best catalytic performance was found for HT3-MW-EcR activated and rehydrated catalyst, with an initial rate of 17.1 × 10^−2^ (mol·l^−1^·h^−1^). On the other, activated catalyst HT3-MW-Ec showed a noteworthy lesser activity with an initial rate of 2.57 × 10^−2^ (mol·l^−1^·h^−1^). The great increase of initial rate may be due to the great hydrophilic character of the hydrotalcite surface in the rehydrated form.

## 4. Conclusion

The Mg-Al-based hydrotalcites with an Mg/Al ratio of 3 were synthesized by the coprecipitation method. The microwave treatments and rehydroxylation of the mixed oxides obtained after calcination were performed. These catalysts were characterized by various physicochemical techniques, which confirmed their crystallinity and purity. The obtained catalysts were studied for the acetone self-condensation reaction in order to evaluate the basicity of these catalysts. It can therefore be concluded that the hydrotalcite compounds were successfully synthesized, and it was shown that the unactivated catalysts give a lower catalytic activity than that found in the case of the calcined catalysts. Since, such self-condensation reactions require a high basicity. In addition, microwave treatment increases the conversion of acetone to diacetone alcohol, and the basicity of the catalysts can also be adjusted by rehydration. Thus, the rehydration of microwave-treated catalyst calcined at 723 K (HT3-MW-EcR) was one of the most active and selective for this reaction and significantly increases the conversion of acetone.

## Figures and Tables

**Table 1:** Main physicochemical characteristics of the catalysts.

Catalysts	*θ*(_003_) (°)	*θ*(_110_) (°)	*d*(_003_) (Å)	*d*(_110_) (Å)	*a*(Å)	*c*(Å)
HT3	5.62	30.24	7.87	1.530	3.061	23.61
HT3-MW	5.56	30.16	7.95	1.534	3.068	23.86
HT3-E	5.66	30.28	7.81	1.528	3.057	23.44
HT3-MW-E	5.61	30.25	7.88	1.530	3.060	23.65

**Table 2:** Values of *β* and *L* for different hydrotalcites synthesized.

Catalysts	*θ*(_003_) (°)	*β*(_003_) (°)	*L*(_003_) (nm)
HT3	5.62	0.5130	15.4
HT3-MW	5.56	0.6158	12.8
HT3-E	5.66	0.4194	18.8
HT3-MW-E	5.61	0.4716	16.7

**Table 3:** Elements analysis results from EDX data for the two catalysts.

Catalysts	Mass (%) of chemical elements		
	O	Mg	Al
HT3-Ec	16.32	54.24	29.44
HT3-MW-Ec	12.84	56.66	30.50

**Table 4:** Specific surface area of the solids by the MB spot-test method.

Catalysts	Specific surface area (m^2^·g^−1^)
HT3-Ec	207 ± 5
HT3-MW-Ec	232 ± 5

**Table 5:** Conversion in (%) of acetone with the catalysts: HT3-Ec and HT3-MW-Ec, as a function of time.

Time (h)	Conversion (%) with HT3-Ec	Conversion (%) with HT3-MW-Ec
2	4.7	5.6
4	7.1	9.5
6	11.0	15.4
8	13.2	18.3

**Table 6:** Initial reaction rate for the conversion of acetone with the catalysts HT3-MW-Ec and HT3-MW-EcR.

Catalysts	*V*_0_ (mol·l^−1^·h^−1^)
HT3-MW-Ec	2.57 × 10^−2^
HT3-MW-EcR	17.1 × 10^−2^

## Data Availability

The data used to support the findings of this study are available from the corresponding author upon request.
